# Effect of Porosity of Alumina and Zirconia Ceramics toward Pre-Osteoblast Response

**DOI:** 10.3389/fbioe.2015.00175

**Published:** 2015-10-28

**Authors:** Chrystalleni Hadjicharalambous, Oleg Prymak, Kateryna Loza, Ales Buyakov, Sergei Kulkov, Maria Chatzinikolaidou

**Affiliations:** ^1^Department of Materials Science and Technology, University of Crete, Heraklion, Greece; ^2^IESL-FORTH, Heraklion, Greece; ^3^Inorganic Chemistry, Center for Nanointegration Duisburg-Essen (CeNIDE), University of Duisburg-Essen, Essen, Germany; ^4^Tomsk State University and ISPMS RAS, Tomsk, Russia

**Keywords:** zirconia, alumina, ceramic mechanical properties, cell adhesion, cell proliferation, porosity, pre-osteoblasts MC3T3-E1

## Abstract

It is acknowledged that cellular responses are highly affected by biomaterial porosity. The investigation of this effect is important for the development of implanted biomaterials that integrate with bone tissue. Zirconia and alumina ceramics exhibit outstanding mechanical properties and are among the most popular implant materials used in orthopedics, but few data exist regarding the effect of porosity on cellular responses to these materials. The present study investigates the effect of porosity on the attachment and proliferation of pre-osteoblastic cells on zirconia and alumina. For each composition, ceramics of three different porosities are fabricated by sintering, and characterized using scanning electron microscopy, energy dispersive X-ray spectroscopy and X-ray powder diffraction. Cell proliferation is quantified, and microscopy is employed to qualitatively support the proliferation results and evaluate cell morphology. Cell adhesion and metabolic activity are found comparable among low porosity zirconia and alumina. In contrast, higher porosity favors better cell spreading on zirconia and improves growth, but does not significantly affect cell response on alumina. Between the highest porosity materials, cell response on zirconia is found superior to alumina. Results show that an average pore size of ~150 μm and ~50% porosity can be considered beneficial to cellular growth on zirconia ceramics.

## Introduction

Zirconia (ZrO_2_) and alumina (Al_2_O_3_) ceramics are among the strongest materials used in medicine. They exhibit outstanding mechanical properties, which make them suitable for load-bearing and wear-resistant applications in bone (Bauer et al., 2013). More than 20 years ago, zirconia and alumina were introduced for total hip arthroplasty (Piconi et al., 2003; Chevalier and Gremillard, 2009). Their clinical success is reflected by the implantation of more than 3.5 million alumina components and more than 600,000 zirconia femoral heads worldwide since 1990 despite some limitations (Chevalier, 2006; Roualdes et al., 2010). Besides the suitability of mechanical properties, the biological response elicited by ceramic materials is also crucial for the clinical success of an implant. Events that take place at the tissue–material interface principally determine implant integration into bone (Masters and Anseth, 2004). Specifically, it is acknowledged that a strong initial attachment of osteoblastic cells or their precursors onto biomaterials leads to better bonding between bone and implant (Anselme, 2000; Kimura et al., 2012). In this respect, several studies have shown that zirconia and alumina ceramics have good biocompatibility (Manicone et al., 2007; Bauer et al., 2013) and show no cytotoxic effects when added to cell cultures, either in monolithic (Josset et al., 1999) or nanopowder forms (Roualdes et al., 2010). Nevertheless, they are generally considered as bioinert materials as they are not capable of creating a biologically relevant interface with bone (Dehestani et al., 2012).

Previous studies on hydroxyapatite (HA) ceramics (Hing, 2005; Lew et al., 2012; Michailidis et al., 2014) as well as on metallic scaffolds of titanium (St-Pierre et al., 2005) and tantalum (Balla et al., 2010) have shown that material bioactivity is affected by the degree of scaffold porosity. An explanation for this is that effective circulation of fluid and transportation of nutrients through a porous structure favor cell migration and proliferation, and lead to better bonding with host tissues. The formation of pores in ceramics broadens their possible applications as they can also be used to deliver biomolecules such as bone morphogenetic proteins (BMPs) with sustained release profiles in the human body (Lew et al., 2012). Within non-resorbable HA scaffolds, a porosity threshold of around 60% exists, below which sustainable bone integration cannot be expected (Hing, 2005). Additionally, a pore size of 100 μm is often considered as a minimum requirement for healthy ingrowth in porous HA, but 300 μm is the optimum size for osteoconduction (Lew et al., 2012).

Such detailed information is not available for either zirconia or alumina ceramics. However, previous *in vivo* experiments indicated that macroporous (pore size >50 nm) alumina allowed the apposition of physiological bone tissue unlike dense alumina implants, which were surrounded by fibrous tissue (Eckert et al., 2000). Other studies have shown that porous alumina coatings improved the mechanical properties of titanium implants, while the pores could be impregnated with bioactive materials, providing a good surface for osteoblastic growth (Karlsson et al., 2003; Walpole et al., 2009). Similarly, a series of studies investigated the use of highly porous zirconia (84–87% porosity) as a substrate for HA coating, which resulted in a strong and bioactive scaffold with good bone regeneration demonstrated *in vivo* (Kim et al., 2008). It was suggested that zirconia had a positive impact on the osteoconductivity of the scaffold in addition to enhancing its mechanical properties. From these studies, it was proposed that even a bioinert ceramic could be used as a substrate material for tissue growth if it had an appropriate architecture and pore characteristics, and therefore further research in this regard was important.

In previous studies, we investigated the osteogenic potential of pre-osteoblasts on porous magnesia and yttria-stabilized zirconia ceramics (Hadjicharalambous et al., 2015b), as well as the pre-osteoblastic cell response on zirconia, alumina, and zirconia/alumina composite (Hadjicharalambous et al., 2015a). The objective of this study was to investigate the effect of zirconia and alumina ceramic substrate porosity on cellular adhesion and proliferation. Ceramics of three different porosities were produced by sintering and characterized regarding porosity, pore size, and phase composition by X-ray powder diffraction (XRD) and energy dispersive X-ray spectroscopy (EDS). The impact of porosity was investigated using MC3T3-E1 pre-osteoblasts by analyzing the metabolic activity of the cells with the PrestoBlue^®^ assay as well as their morphology on the different substrates by SEM.

## Materials and Methods

### Ceramic Fabrication and Characterization

Alumina and zirconia ceramics with three porosities (A, B, and C from smaller to larger porosity) were fabricated for the experiments. Starting powders of Al_2_O_3_ or ZrO_2_ stabilized with 3 mol% yttrium oxide or yttria Y_2_O_3_ (Siberian Enterprise Chemical Group, Russia) were used. Pure zirconia undergoes phase transformation from the tetragonal to the monoclinic phase during sintering; this process occurs with a volume change, leading to sudden failure of the material when zirconia cools. ZrO2 stabilized with yttria can maintain its tetragonal phase at room temperature and is the principal kind of zirconia considered for current medical use (Manicone et al., 2007). Briefly, the ceramic powders were cold pressed on a hydraulic press under 100 MPa pressure in steel die molds in order to obtain cylindrical (15 mm in diameter, 5 mm in height) forms. To create porosity, organic material particles (polyethylene) were added as pore formers into the powder mixtures. The size range of the porogens was 50–150 μm with a mean size of 100 μm for 49 and 63% porosity samples, and 30–120 μm with a mean size of 75 μm for lower porosity samples.

The compacted powder samples were sintered in air at a peak temperature of 1350°C (for 49 and 63% porosity), 1400°C (for 30 and 34% porosity), and 1450°C (for 23 and 24% porosity) in LHT 02/17 High-Temperature Furnaces (Nabertherm) with an isothermal exposure time of 1 h. During thermal treatment, the organic material was extracted, generating the desired pores within the microstructure.

The porosity of each sample was calculated by dividing the scaffold density (ρ_scaffold_) by the theoretical material density (ρ_material_), and subtracting the result from one (Karageorgiou and Kaplan, 2005; Galmarini, 2011):

$$P_{\text{total}}=(\text{1}-r_{\text{scaffold}}/r_{\text{material}})\;\text{x}\;100$$

The scaffold density was determined by dividing the weight by the volume of the scaffold and the material density is the density of the material of which the scaffold is fabricated (specifically, for alumina samples: ρ_material_ = 3.99 g cm^−3^; and for zirconia samples: ρ_material_(Zr-A) = 5.84, ρ_material_(Zr-B) = 5.88, ρ_material_(Zr-C) = 5.90 g cm^−3^ as calculated based on their monoclinic and tetragonal phase compositions shown in Table 1). The average pore size was measured for each of the three porosity types through analysis of scanning electron microscopy images (Philips SEM-515).

**Table 1:** **Pore sizes and porosities of zirconia and alumina ceramics**.

Sample	Chemical composition	Porosity (%)	Small pore mean size (**μ**m)	Large pore mean size (**μ**m)
Zr-A	(Zr,Y)O_1.95_, ZrO_2_	23	3.1	–
Zr-B	(Zr,Y)O_1.95_, ZrO_2_	30	6.8	–
Zr-C	(Zr,Y)O_1.95_, ZrO_2_	49	0.7	167 ± 113
Al-A	Al_2_O_3_	24	3.4	–
Al-B	Al_2_O_3_	34	2.2	–
Al-C	Al_2_O_3_	63	2.1	141 ± 113

X-ray powder diffraction was performed with a Bruker D8 Advance X-ray diffractometer in Bragg–Brentano mode with Cu Kα radiation (λ = 1.5418 Å; 40 kV and 40 mA). The ceramic samples were investigated in the range of 10–90° 2θ with a step size of 0.01° 2θ and a counting time of 0.6 s. Rietveld refinement with the TOPAS 4.2 program package from Bruker was performed in order to analyze the crystallographic properties of the samples. In this way, the weight amount of crystalline phases, their lattice parameters and percentage of yttrium substitution in the yttria-stabilized zirconia, as well as the average crystallite size and the crystallographic density were determined. The patterns of rhombohedral Al_2_O_3_ (#043-1484, corundum), monoclinic ZrO_2_ (#83-0940), and tetragonal phase Zr_0.9_Y_0.1_O_1.95_ (#82-1241) from the ICDD database were used as reference for the qualitative phase analysis, which was performed with a Diffrac.Suite EVA V1.2 from Bruker. For each Rietveld refinement, the instrumental correction, as determined with a standard powder sample LaB_6_ from NIST (National Institute of Standards and Technology) as standard reference material [SRM 660b; a(LaB_6_) = 4.15689 Å], was taken into account.

For the morphological characterization of the ceramic samples, scanning electron microscopy was performed on a FEI Quanta 400 ESEM instrument in high vacuum after sputtering with Au/Pd (80:20). Energy-dispersive X-ray spectroscopy (EDS) with an accelerating voltage of 15 kV was carried out with a Genesis 4000 instrument with SUTW-Si(Li) detector.

### Cell Culture and Reagents

Minimum essential Eagle’s medium (α-MEM), penicillin/streptomycin, fetal bovine serum (FBS), and trypsin/EDTA were purchased from Sigma (St. Louis, MO, USA). PrestoBlue^®^ reagent for cell viability was purchased from Invitrogen Life Technologies (Carlsbad, CA, USA) and cell culture plates from Corning.

The MC3T3-E1 murine pre-osteoblastic cells (Beck et al., 1998) were cultured in α-MEM medium supplemented with 10% fetal bovine serum (FBS) and 1% penicillin/streptomycin (primary medium) and maintained at 37°C in a humidified atmosphere of 5% CO_2_ in air. Cells were splitted once a week with trypsin/EDTA. Confluent cells were harvested using trypsin/EDTA, counted on a hemocytometer, and seeded onto the ceramic samples. For all experiments, cells between passage 6 and 15 were used.

### Cell Culture on Porous Ceramics

Ceramic sample preparation and the cell seeding procedure were performed as previously described (Hadjicharalambous et al., 2015b). Cells (5 × 10^4^ cells) were seeded onto the samples in a 30 μL cell suspension in primary medium. The medium was replaced with fresh medium every 2 days.

### Cell Proliferation Assay

The PrestoBlue^®^ assay (Invitrogen, CA, USA) was used to monitor proliferation of MC3T3-E1 pre-osteoblasts after 2, 4, and 8 days of culture. PrestoBlue^®^ assay is dependent on the cellular reduction of a blue colored, cell permeant, resazurin-based compound by viable cells to a red product, which can be detected spectrophotometrically and provides a measure of cell viability. Assessment of proliferation at each time point was performed as described in Hadjicharalambous et al. (2015b). For each ceramic surface, three replicates were used (*n* = 3). Data from three independent experiments were averaged as mean values ± SEM for each time point and sample. Statistical analysis was performed using ANOVA (GraphPad Prism 5 software) to evaluate the differences among ceramic samples. A *p* value of <0.05 was considered significant.

### Scanning Electron Microscopy

The morphology of adherent MC3T3-E1 cells was assessed by scanning electron microscopy (SEM). Cells (5 × 10^4^ cells/sample) were cultured on alumina and zirconia substrates for 1 or 10 days as described above, rinsed with 0.2 M sodium cacodylate buffer and fixed in 2% glutaraldehyde and 2% paraformaldehyde for 1 h, at 4°C. Cells were then post fixed in 1% osmium tetroxide for 30 min at 4°C and dehydrated through a series of increasing concentrations of ethanol (from 30 to 100%) and dried by applying critical drying with CO_2_ at 33°C and 73 atm (Baltec CPD 030). Following sputtering with a 20 nm thick layer of gold (Baltec SCD 050), ceramic samples were observed under a scanning electron microscope (JEOL JSM-6390 LV) with an accelerating voltage of 15 kV.

### Fluorescence Microscopy

Living cells on the ceramic samples were fluorescently labeled with carboxyfluorescein diacetate succinimidyl ester (CFSE) (Invitrogen, CA, USA). One hundred thousand cells were cultured on the ceramic substrates for 1 or 7 days. At the end of incubation, the ceramics were washed once with PBS, pH 7.4 and cells were then stained with 20 μm CFSE dye in PBS for 15 min and viewed by fluorescence microscopy (496ex/516em).

## Results

### Ceramic Characterization

**Figure 1:**
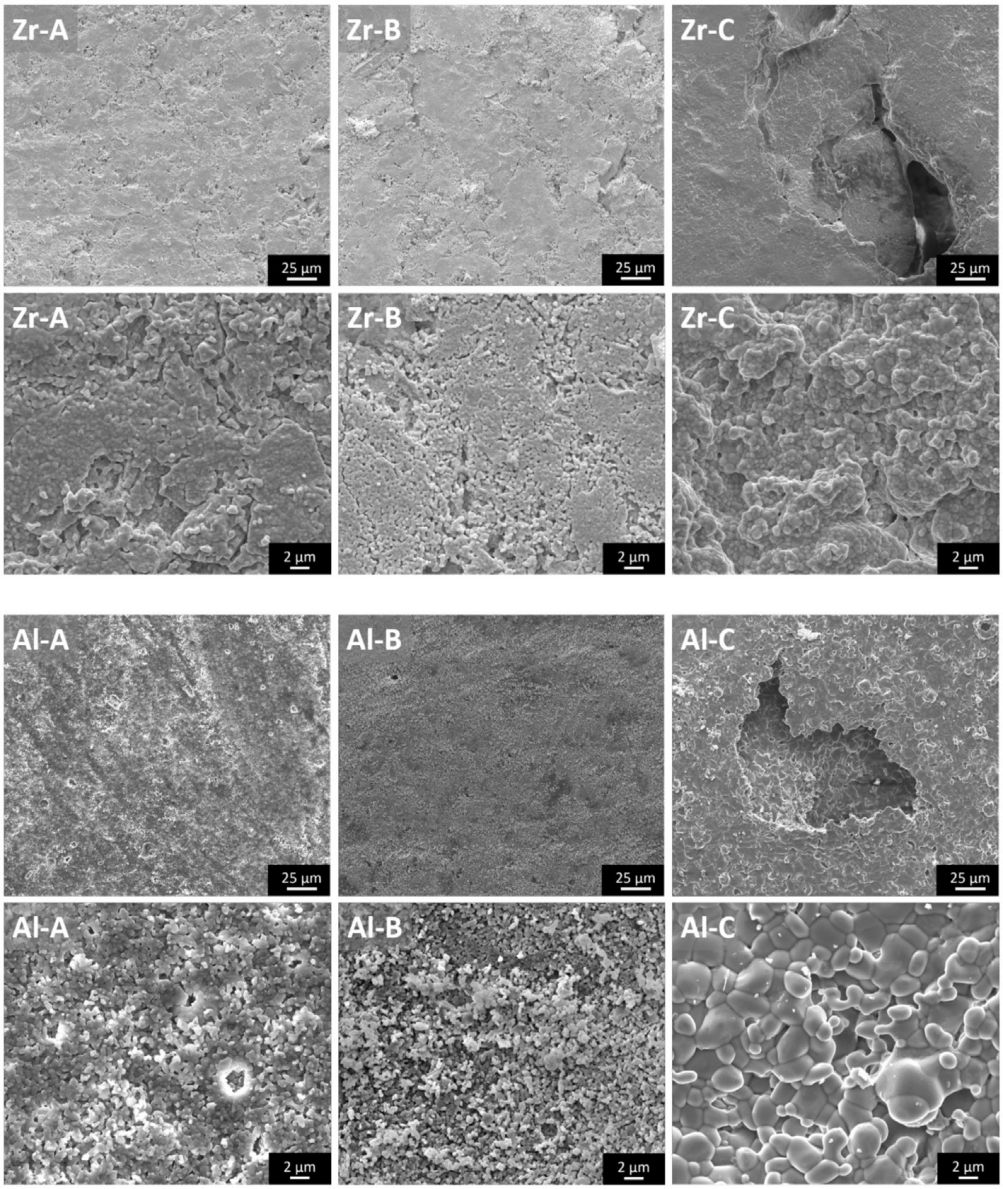
**The microstructure of zirconia and alumina ceramics as shown by scanning electron microscopy images**. The large pores of nanostructural Zr-C and microstructural Al-C ceramics are visible. Original magnifications are ×1000 for larger and ×8000 for smaller scale bar images.

Pore size and porosity characteristics of zirconia and alumina ceramics are provided in Table 1. Figure 1 shows the surface morphology of alumina and zirconia ceramics with different porosities A, B, and C, as investigated by SEM. According to the microscopic characterization, the low porosity ceramics (A and B) contain only small pores (<6 μm on average), whereas the higher porosity Al-C and Zr-C samples contain small as well as large pores with average size of 141 and 167 μm, respectively, as shown in Table 1. The visible grain size of alumina ceramics was larger in comparison to zirconia samples, and in accordance with the XRD results (Table 2).

**Table 2:** **Crystallographic properties of alumina and yttria-stabilized zirconia as determined by Rietveld refinement**.

Sample	Phases	Wt. %	Density (g cm^−3^)	Lattice parameters				Volume of unit cell	Crystallite sizes (nm)

				*a*/Å	*b*/Å	*c*/Å	β/°	*V*/Å^3^	
Al-A	Al_2_O_3_	100	3.99	4.759 (1)	–	12.996 (1)	–	254.94 (1)	>500
Al-B	Al_2_O_3_	100	3.99	4.759 (1)	–	12.995 (1)	–	254.92 (1)	>500
Al-C	Al_2_O_3_	100	3.98	4.761 (1)	–	12.999 (1)	–	255.19 (1)	>500
Zr-A	ZrO_2_(monoc.)	59	5.75	5.176 (1)	5.225 (1)	5.326 (1)	99.10	142.25 (3)	68
	Zr_0.85_Y_0.15_O_1.95_	41	5.97	3.622 (3)	–	5.174 (1)	–	67.88 (2)	63
Zr-B	ZrO_2_ (monoc.)	47	5.76	5.176 (1)	5.222 (1)	5.325 (1)	99.11	142.12 (2)	56
	Zr_0.87_Y_0.13_O_1.95_	53	5.98	3.618 (1)	–	5.178 (1)	–	67.77 (1)	105
Zr-C	ZrO_2_ (monoc.)	49	5.78	5.164 (1)	5.212 (1)	5.327 (1)	99.05	141.59 (3)	76

	Zr_0.89_Y_0.11_O_1.95_	51	6.01	3.616 (1)	–	5.176 (1)	–	67.66 (2)	59
Bondars et al. (1995)	ZrO_2_ (tetrag.)		6.10	3.596 (1)	–	5.184 (1)	–	67.04 (1)	–

**Figure 2:**
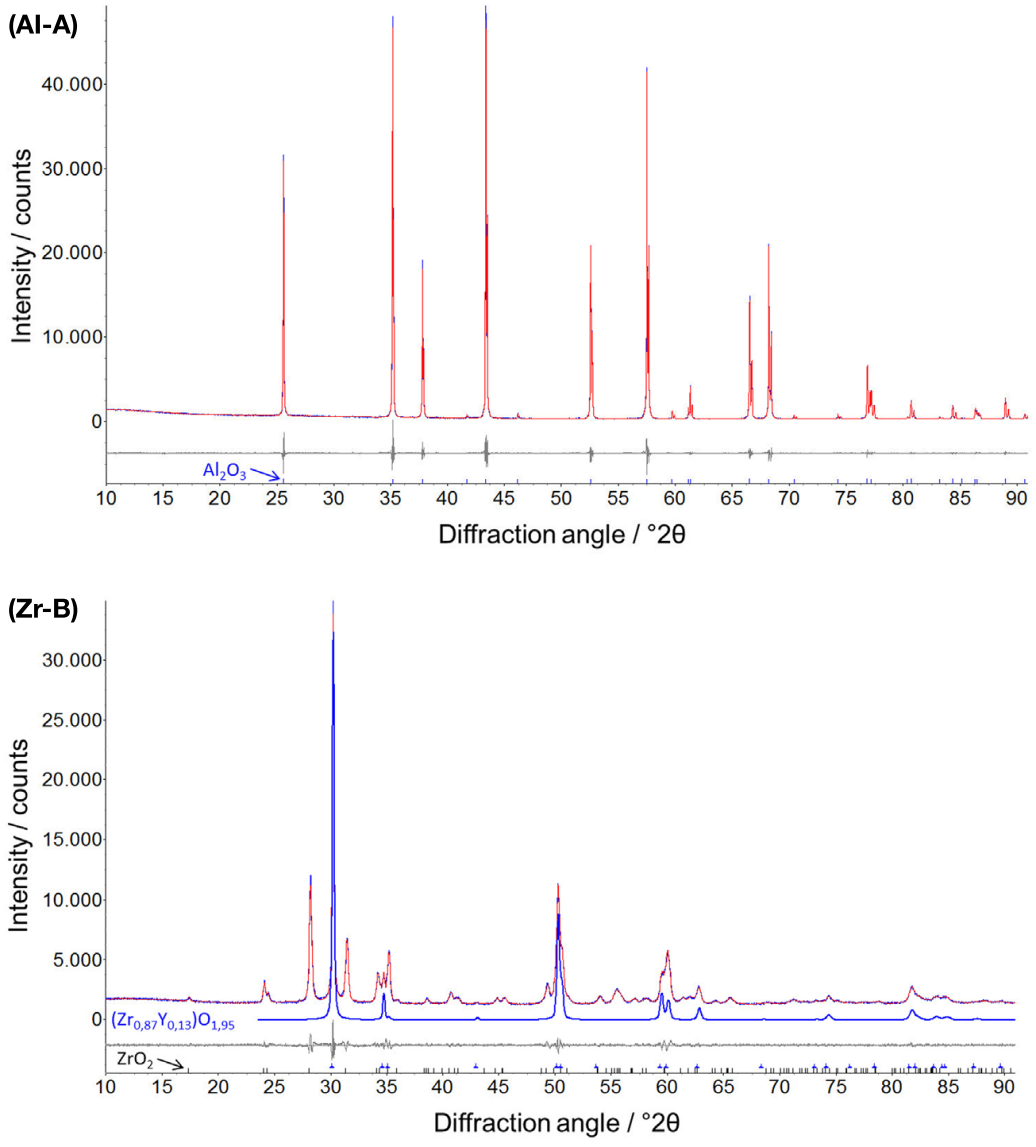
**Representative X-ray powder diffractograms of alumina (Al-A, Rwp **=** 5.5) and yttria-stabilized zirconia (Zr-B, Rwp **=** 3.5) with additionally denoted rhombohedral Al_2_O_3_ (corundum) and tetragonal Zr_0.87_Y_0.13_O_1.95_ phases**. Profile and difference plots from Rietveld refinement are shown.

The ceramic samples with different porosities were characterized by XRD and two representative diffractograms for alumina (Al-A, 24% porosity) and yttria-stabilized zirconia (Zr-B, 30% porosity) are depicted in Figure 2. It was shown that all alumina ceramics consist only of rhombohedral Al_2_O_3_ phase (corundum), whereas the yttria-stabilized zirconia ceramics consist of a two phase system of both monoclinic ZrO_2_ phase and tetragonal phase Zr_0.87_Y_0.13_O_1.95_ in approximately equal amounts.

By means of Rietveld refinement, the crystallographic properties of the investigated ceramics were determined (Table 2). The calculated lattice parameters and the resulting crystallographic densities for the corundum phase were very similar among alumina ceramics, confirming the phase stability in all samples. The determined crystallite size of Al_2_O_3_ phases, using the Scherrer equation (Scherrer, 1918) were in the μm-region, which can also be confirmed by the corresponding sharp peaks in the diffractogram (Figure 2, Al-A). In contrast to alumina, the yttria-stabilized zirconia samples exhibited nanocrystalline phases with the calculated crystallite size being approximately 60–100 nm. The presence of smaller crystallites (a crystallite is part of one grain) within yttria-stabilized zirconia, in comparison to larger crystallites in alumina, was also confirmed by scanning electron microscopy (Figure 1). No significant dependence of the crystallite size upon the porosity of ceramic samples could be detected.

Using Rietveld refinement and comparing the calculated volumes of the unit cells for Zr-containing phases (Table 2), it was possible to estimate the percent substitution of smaller Zr atoms (159 pm) by larger Y atoms (180 pm). It was shown that with increasing porosity, the volume of the tetragonal unit cell (Zr,Y)O_1.95_ decreases slightly, whereas the volume of the monoclinic ZrO_2_ unit cell (no significant substitution) remains almost unchanged. In this way the amount of incorporated Y-atoms into the tetragonal phase was determined as 15, 13, and 11 mol% for the 23, 30, and 49% porous zirconia, respectively (Table 2). With the calculated weight percentage of the monoclinic ZrO_2_ and tetragonal (Zr,Y)O_1.95_ phases and defined site occupancy of Zr/Y-atoms in the corresponding unit cells, about 4.5 wt.% of Y could be determined crystallographically in the yttria-stabilized zirconia.

**Figure 3:**
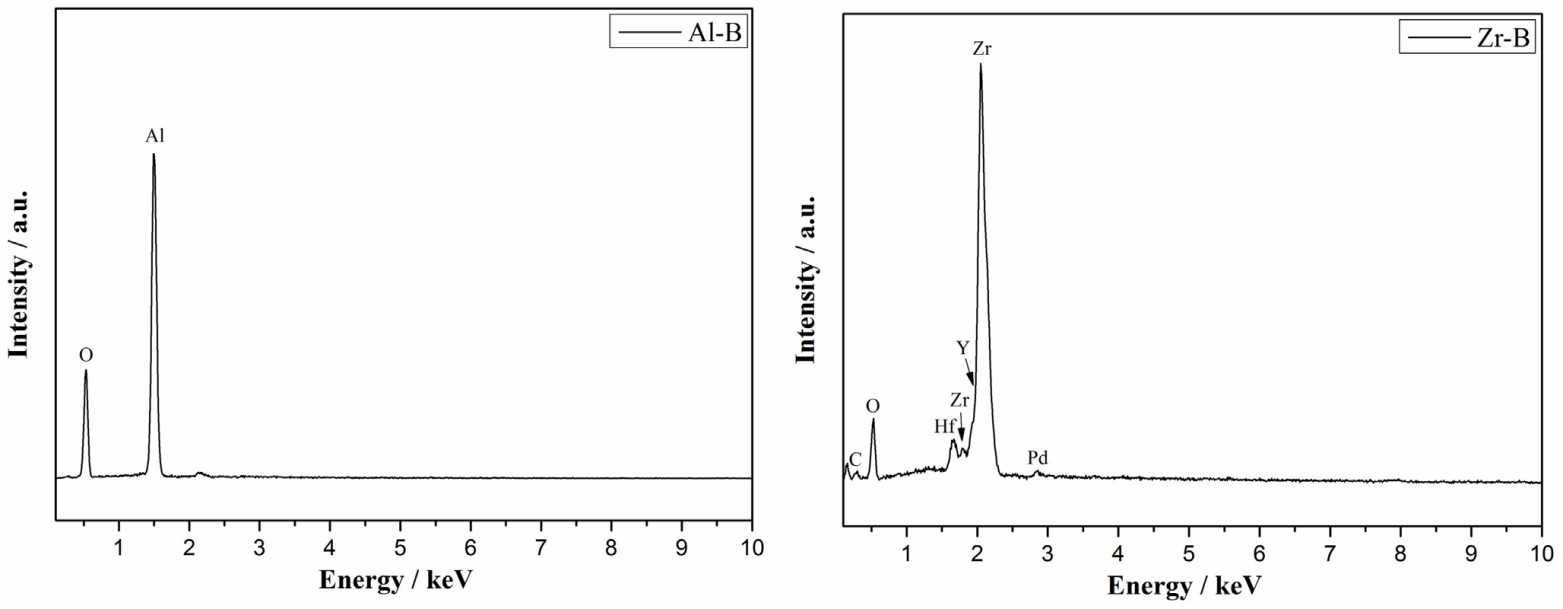
**Representative EDS analysis showing the composition of alumina (Al-B, porosity 34%) and yttria-stabilized zirconia (Zr-B, porosity 30%)**.

A good correlation between the above results and the EDS quantitative analysis of chemical elements was found (Figure 3). In alumina samples only the Al (64 wt.%) and O (35 wt.%) elements were found, confirming the composition of corundum phase. In yttria-stabilized zirconia samples, the Zr (64 wt.%), Y (8 wt.%), O (13 wt.%) elements were identified in addition to Hf (10 wt.% or 3 at.%), which was present as a natural impurity of ZrO_2_ ceramics (Wang et al., 2010). Notably, a decreasing amount of yttrium was detected with increasing sample porosity and specifically 8.3, 7.9, 7.5 wt.% of yttrium for Zr-A, Zr-B, and Zr-C, respectively. A similar effect was also observed by XRD.

**Figure 4:**
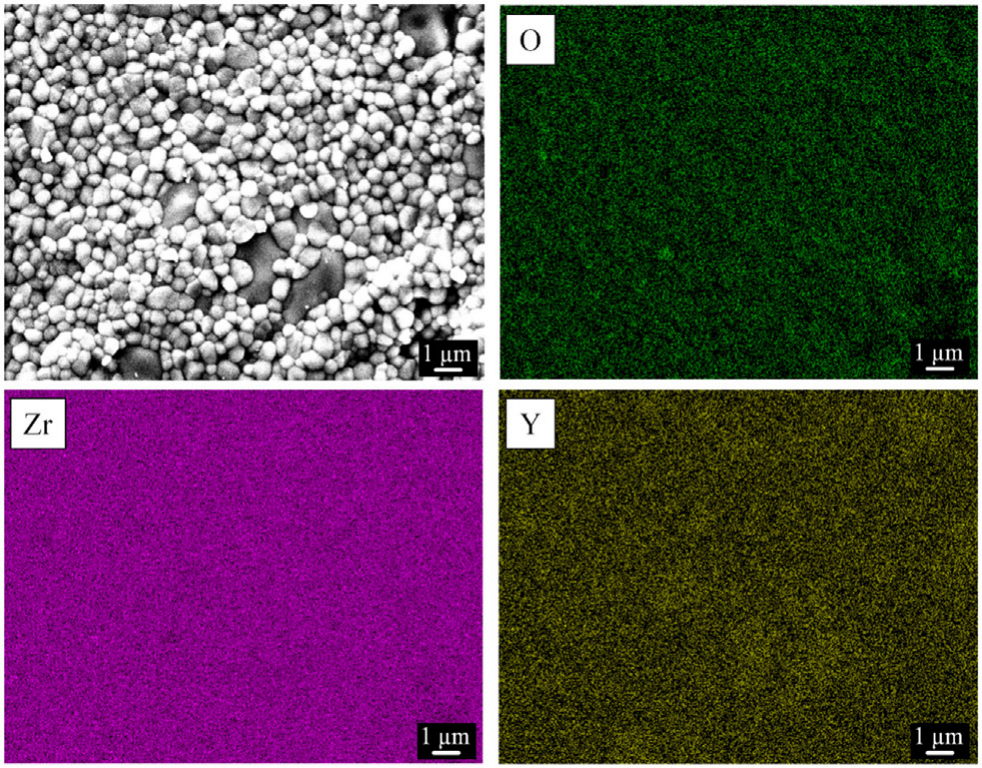
**Representative elemental mapping of yttria-stabilized zirconia (Zr-C, porosity 49%) by energy-dispersive X-ray spectroscopy**. A corresponding scanning electron microscopy image of the investigated surface is shown.

Figure 4 shows the EDS maps of oxygen, zirconium, and yttrium in yttria-stabilized zirconia. The images indicate a regular distribution of the three elements in the ceramic sample, confirming in this way a homogeneous distribution of the crystalline monoclinic ZrO_2_ and tetragonal (Zr,Y)O_1.95_ phases and as a result, a good substitution of Zr by Y atoms. These results were confirmed in all three zirconia samples.

### Cell Metabolic Activity

**Figure 5:**
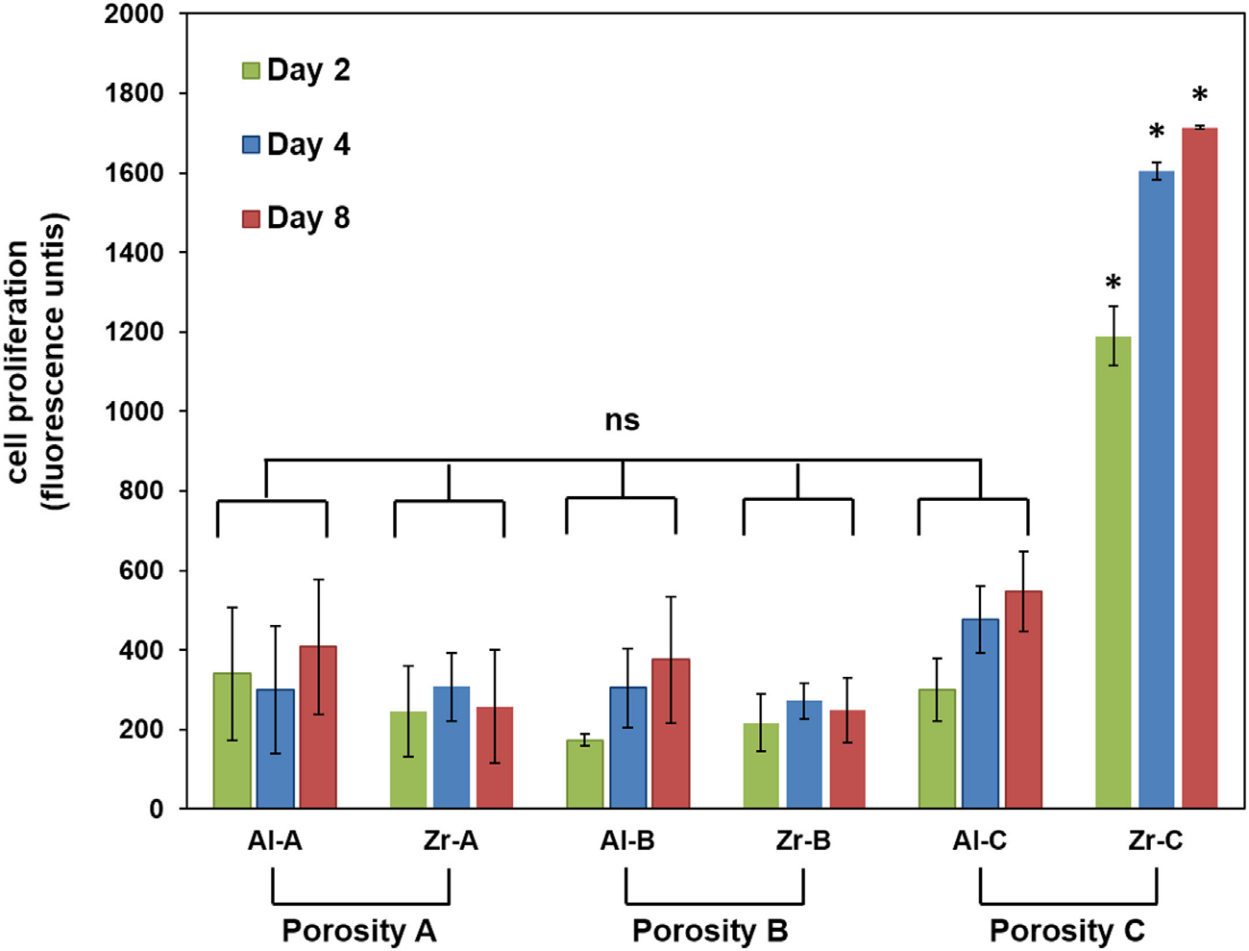
**Cell proliferation by PrestoBlue^®^ assay for 24% (Al-A), 34% (Al-B), and 63% (Al-C) porous Al_2_O_3_ and 23% (Zr-A), 30% (Zr-B), and 49% (Zr-C) porous ZrO_2_ substrates up to 8 days of culture**. The metabolic activity of pre-osteoblasts on the highest porosity (49%) zirconia samples was significantly higher (about threefold) compared to other samples at all time points (*p < 0.05, *n* = 9). All other samples showed non-significant (ns) differences in cellular growth among them.

The PrestoBlue^®^ assay was used to quantitatively determine the proliferation of viable MC3T3-E1 cells on porous zirconia and alumina substrates. A comparison of the cellular metabolic activity on the different samples after 2, 4, and 8 days of culture is depicted in Figure 5. Pre-osteoblasts displayed similar metabolic activities on Al-A, Al-B, Zr-A, and Zr-B porous ceramics, regardless of chemistry or porosity, and no significant differences between these substrates were observed. Among zirconia substrates, cell densities were found significantly higher on the highest porosity sample Zr-C for all culture time points. Among alumina, improved metabolic activity was observed on Al-C, but this was not significantly higher than on Al-A or Al-B samples.

**Figure 6:**
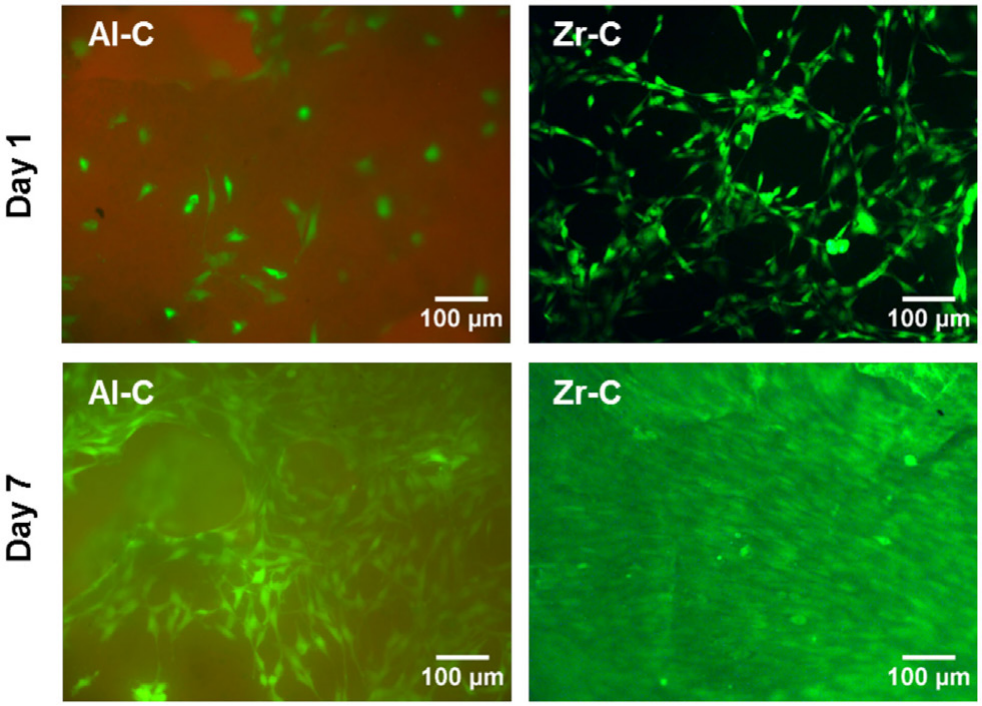
**Fluorescent live cell staining of MC3T3-E1 cells cultured for 1 day (upper panel) and 7 days (lower panel) on 63% porous alumina (Al-C, left) and 49% porous zirconia (Zr-C, right) ceramics**. Original magnification is ×10. After 7 days of culture, cell growth was observed and higher cell densities were evident on zirconia than alumina. Scale bar represents 100 μm.

Fluorescence microscopy was also employed to qualitatively observe live CFSE-stained cells on the ceramic samples, and assess their growth. As depicted in Figure 6, cell proliferation was observed on both Al-C and Zr-C after 7 days of culture, but was evidently higher on Zr-C, as shown by the formation of a uniform layer of green fluorescing cells on this material. Cells on lower porosity ceramics were also stained with CFSE in an attempt to monitor changes in living cell numbers, however, it was not possible to record clear images of cells on the samples (data not shown), due to strong background fluorescence interference coming from the samples themselves.

### Cellular Attachment and Morphology

**Figure 7:**
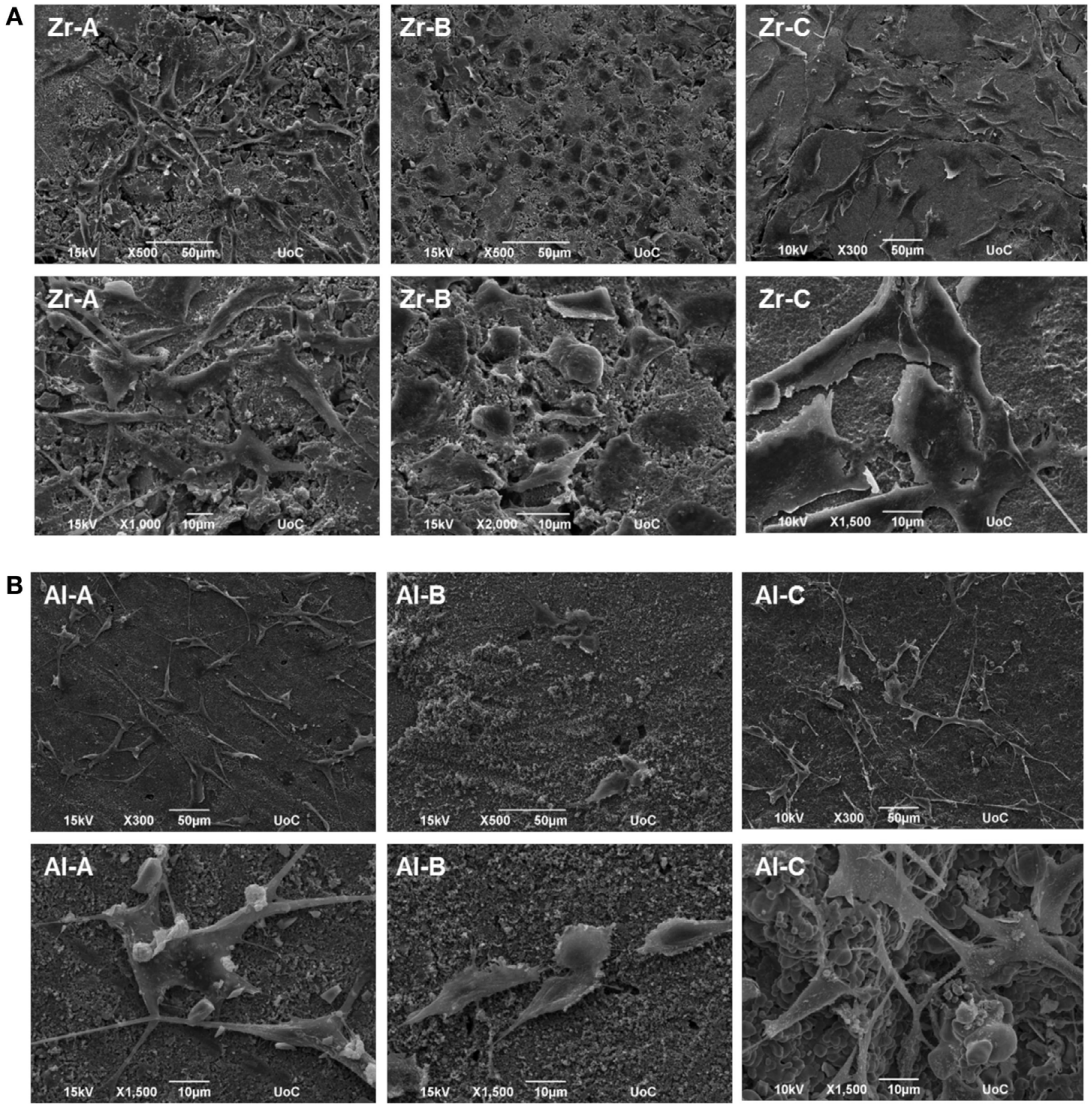
**Scanning electron microscopy (SEM) images showing morphology of MC3T3-E1 cells after day 1, on (A) zirconia of 23% (Zr-A), 30% (Zr-B), and 49% (Zr-C) porosities or (B) alumina of 24% (Al-A), 34% (Al-B), and 63% (Al-C) porosities**. Increasing porosity resulted in better cell spreading on zirconia but not on alumina ceramics, where cells mostly displayed a long and spindled morphology. Original magnifications are ×500 or ×300 for upper, and ×1000 to ×2000 for lower images in **(A,B)**.

Pre-osteoblastic cell morphology on the different porous alumina and zirconia ceramic substrates was investigated by scanning electron microscopy. Figure 7 shows the morphologies of the cells on samples after 1 day of culture. The results showed that cellular appearance and density strongly depended on the substrate. Between zirconia and alumina, cell adhesion morphology was more flattened on zirconia. Specifically, cells adherent on Zr-A or Al-A were found to exhibit branched morphology characterized by long spindle-like cellular extensions, attaching to the sub-micrometer features of the ceramics as well as nearby cells. In contrast, cells cultured on either 30% porous zirconia (Zr-B) or 34% porous alumina (Al-B) appeared small and round-shaped with under-developed filopodia, whereas cell density appeared to be higher on Zr-B. A further increase in the porosity of zirconia ceramics resulted in a profound effect on cell adhesion, with cells exhibiting flattened morphology and very good membrane spreading on the 49% porous zirconia (Zr-C) substrate (Figure 7A). Interestingly, increasing porosity in alumina ceramics to 63% (Al-C) had no evident impact on cell morphology, as cell spreading on the substrates was limited and spindled morphology was dominant (Figure 7B).

**Figure 8:**
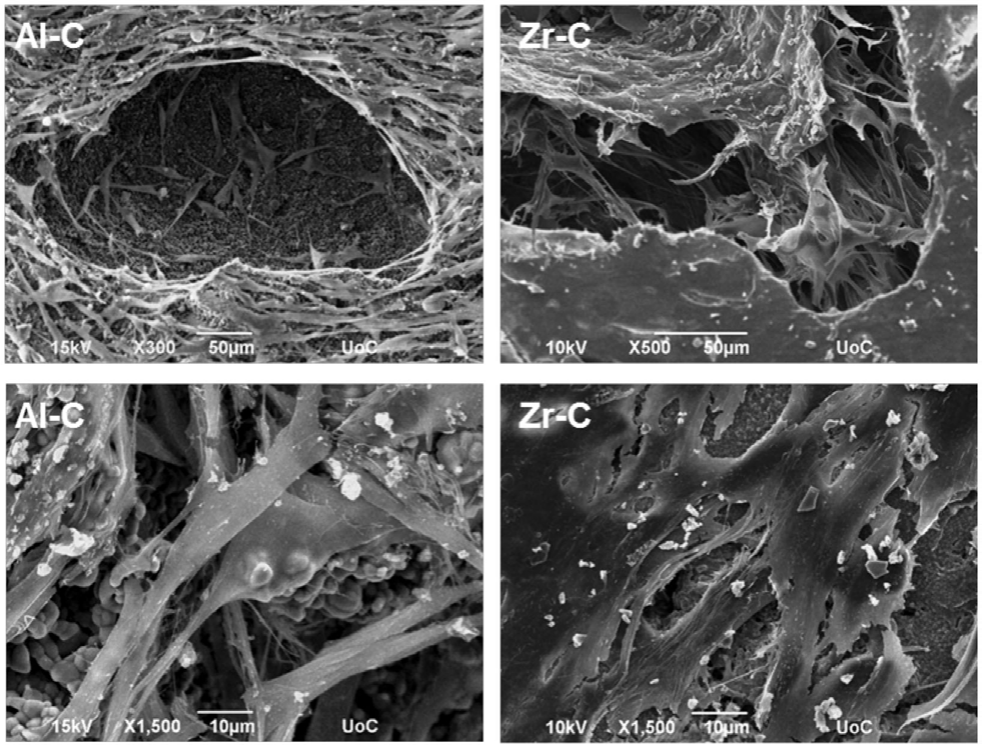
**Scanning electron microscopy (SEM) images showing cell morphology of MC3T3-E1 cells after 10 days, on 49% porous zirconia (Zr-C, right) or 63% porous alumina (Al-C, left) ceramics**. A smoother, denser multilayer spreading over pore openings was observed on zirconia. Original magnifications are ×500 and ×300 for upper, and ×1500 for lower images.

Cellular growth on the Al-C and Zr-C ceramic samples after 10 days of culture was also assessed by SEM. As shown in Figure 8, proliferation of the MC3T3-E1 pre-osteoblastic cells occurred on both Al-C and Zr-C samples. However, cells on Zr-C formed a dense layer that could also bridge large pore openings, unlike cells cultured on Al-C. In addition, the cell matrix on Zr-C appeared more uniform as individual cells could not always be identified (SEM observations), contrary to cells on Al-C, which maintain their initial spindle-shaped morphology and were easily distinguished.

## Discussion

In recent years, it has been recognized that biomaterial porosity greatly influences cellular behavior not only at the proliferative but also the differentiation stage (Bignon et al., 2003; Karageorgiou and Kaplan, 2005; Lew et al., 2012). Therefore, it has been suggested that by adapting surface properties to the desired cell behavior, we may open up the possibility of controlling cell behavior, thereby improving implant performance (Ni et al., 2014).

The present *in vitro* cell–material interaction study clearly indicates that porosity is an important parameter regarding cell adhesion and growth on ceramic materials, as assessed both qualitatively and quantitatively by employing microscopy and cell viability methods, respectively. Enhanced cellular response in terms of adhesion density (qualitative observation, as shown in Figure 7) and proliferation of pre-osteoblasts, was observed when the porosity of zirconia increased from 23 to 49%, with the simultaneous introduction of pores of approximately 55–280 μm (average size 150 μm), which are presumed to have a positive impact on cell growth (Lew et al., 2012).

Cell adhesion and proliferation also depend on material chemistry (Hing, 2005). In this study, alumina and zirconia ceramics did not exhibit significant differences in cellular growth or adhesion, when porosity was low (samples with A and B porosities).

Though material chemistry can be a determinant factor in cell–material interactions, metal oxides such as alumina and zirconia are generally considered bioinert. Their particles (at 2 μm size) have been reported not to be toxic to osteoblasts (Roualdes et al., 2010), whereas their ionic forms of Zr^4+^ and Al^3+^ exhibit low to medium toxicity, but such ionic forms are present only at low pH (Franks and Gan, 2007).

In a previous report (Lohmann et al., 2002), higher osteoblast proliferation was observed in the presence of zirconia than in the presence of alumina particles, an effect that the authors found to be related to the higher reactive surface of the alumina particles, which were significantly smaller than the zirconia ones. However, in our study we used sintered ceramics in which alumina particles on the material surface were larger than zirconia particles for all porosities, as shown by SEM (Figure 1). Considering this, the higher proliferation we observed on porous zirconia cannot be explained on the basis of crystallite size, since the crystallite size of the zirconia ceramics was smaller than the crystallite size of alumina. Hence, it appears that chemical composition or surface topography differences (due to larger crystallite size in alumina ceramics) alone are not sufficient to induce a differential pre-osteoblast adhesion and growth among alumina and zirconia ceramics of low porosities (porosities A and B).

On the other hand, we found that the introduction of larger pores is critical to significantly enhance both cell adhesion and proliferation in zirconia ceramics. This results in higher interconnection between the smaller pores inside the Zr-C sample, and as a consequence, in better circulation of nutrient medium for the cells. The increased total surface of interconnected pores (small and large) in the Zr-C ceramic provides better conditions for a significantly higher cell adhesion (Figures 5–7) and growth within Zr-C. Similarly, a recent study (Song and Cho, 2014) reported better pre-osteoblast spreading and growth on a porous zirconia scaffold (pores between 200 and 400 μm) in comparison to a non-porous zirconia disk. In addition, the authors showed that cell spreading on porous zirconia was better compared to spreading on a porous zirconia scaffold coated with HA. A limitation in their comparison was partial obstruction of the pores caused by the coating, however the importance of substrate architecture (pores) in cell behavior on zirconia ceramics was highlighted.

In this study, we observed better cell adhesion and spreading followed by greater osteoblast proliferation on zirconia (49% porosity) than on alumina (63% porosity) porous scaffolds of similar pore size (porosity C). More flattened cell adhesion on Zr-C than on Al-C on day 1 was followed by the formation of a smoother, more uniform extracellular matrix on Zr-C, as incubation time progressed. Cell survival is dependent on the transformation from spherical to spread-out shape (Andersen et al., 2005) and initial cell adhesion is essential to subsequent proliferation (Anselme, 2000). As far as cell proliferation and differentiation are concerned, a flattened, fully spread cell morphology was reported to be better (Xia et al., 2011). The initially observed flatter cell morphology on Zr-C may serve as a possible explanation as to why Zr-C elicits higher proliferation versus Al-C.

Also, according to the SEM micrographs of the ceramic samples, a greater number of large and deep pores was observed on the surface of Zr-C scaffolds in comparison to Al-C (qualitative observation), which may have contributed to the differences in cell proliferation observed between the highest porosity alumina and zirconia scaffolds.

## Conclusion

Enhancement of cellular response regarding the adhesion and proliferation of MC3T3-E1 pre-osteoblasts was observed by increasing substrate porosity in zirconia ceramics. Our results show that ~50% porosity and an average pore size of 150 μm is beneficial to cellular growth. This suggests an important advantage for porosity in zirconia ceramics, rendering an otherwise bioinert material into a cell-supporting scaffold. Unlike zirconia, cell proliferation on alumina was not significantly improved with increasing porosity. Finally, porous zirconia was found superior to porous alumina as it favored better cell spreading, pore infiltration, and higher growth.

## Conflict of Interest Statement

The authors declare that the research was conducted in the absence of any commercial or financial relationships that could be construed as a potential conflict of interest.
